# Dual Ion Releasing Nanoparticles for Modulating Osteogenic Cellular Microenvironment of Human Mesenchymal Stem Cells

**DOI:** 10.3390/ma14020412

**Published:** 2021-01-15

**Authors:** Yu-Jin Kim, Jaeyoung Lee, Gwang-Bum Im, Jihun Song, Jiwoo Song, Jiyong Chung, Taekyung Yu, Suk Ho Bhang

**Affiliations:** 1 School of Chemical Engineering, Sungkyunkwan University, Suwon 16419, Korea; yujinkim1003@gmail.com (Y.-J.K.); lki1005@skku.edu (G.-B.I.); jih2616@naver.com (J.S.); 2 Department of Chemical Engineering, Kyung Hee University, Youngin 17104, Korea; skvneu30@gmail.com (J.L.); jiwoo715@naver.com (J.S.); 3 BK21 FOUR Integrated Engineering Program, Department of Chemical Engineering, Kyung Hee University, Youngin 17104, Korea

**Keywords:** zinc-based iron oxide nanoparticles, zinc ion, iron ion, mesenchymal stem cells, osteogenic differentiation, angiogenesis

## Abstract

In this study we developed a dual therapeutic metal ion-releasing nanoparticle for advanced osteogenic differentiation of stem cells. In order to enhance the osteogenic differentiation of human mesenchymal stem cells (hMSCs) and induce angiogenesis, zinc (Zn) and iron (Fe) were synthesized together into a nanoparticle with a pH-sensitive degradation property. Zn and Fe were loaded within the nanoparticles to promote early osteogenic gene expression and to induce angiogenic paracrine factor secretion for hMSCs. In vitro studies revealed that treating an optimized concentration of our zinc-based iron oxide nanoparticles to hMSCs delivered Zn and Fe ion in a controlled release manner and supported osteogenic gene expression (RUNX2 and alkaline phosphatase) with improved vascular endothelial growth factor secretion. Simultaneous intracellular release of Zn and Fe ions through the endocytosis of the nanoparticles further modulated the mild reactive oxygen species generation level in hMSCs without cytotoxicity and thus improved the osteogenic capacity of the stem cells. Current results suggest that our dual ion releasing nanoparticles might provide a promising platform for future biomedical applications.

## 1. Introduction

Our body contains various metal ions such as sodium [1,2], magnesium [3], iron (Fe) [4], and zinc (Zn) [5] with specific roles in the body. Since various diseases such as hyponatremia, hyperkalemia, edema, and neurodegenerative disorders are caused by ion imbalances, various biological reactions occur in the body to control the concentrations of the ions [6]. Previous studies have been conducted to control the cellular behaviors through ions. However, direct delivery of ions to the cells usually accompanies high cytotoxicity [7,8,9,10,11].

Many researchers proposed various methods that can indirectly deliver ionic substance into cells using nanoparticles [12,13,14,15,16,17]. Inorganic nanoparticles (NPs) synthesized for dual ions delivery were designed to undergo degradation under low pH-condition. Since the endosomes which capture NPs have a low pH environment (4~5), the NPs after endocytosis can be exposed to low pH condition and undergo degradation into ions [13]. Based on the degradation property of NPs under low pH condition, we tried to deliver representative metal ions such as Zn and Fe for advanced osteogenic differentiation of stem cells. Intracellular delivery of Fe ions is known to induce mild reactive oxygen species (ROS) in and stimulate the secretion of angiogenesis paracrine factors such as vascular endothelial growth factor (VEGF) [14,15]. The expression of representative genes for osteogenic differentiation such as *RUNX2*/*OSTERIX* has been reported to show upregulation with Zn ion treatment [16]. In contrast to previous studies focused on delivering a single type of ion to the cells, we attempted to delivery hetero types of ions to the stem cells with an NP simultaneously.

We applied our NPs to mesenchymal stem cell (MSC), which is one of the most widely studied stem cells for bone regeneration. MSCs have been reported to promote bone regeneration through paracrine factor secretion and direct osteogenic differentiation [18]. In order to increase the efficiency of bone regeneration with MSCs, additional strategies for inducing angiogenesis are required [19]. Nevertheless, no method has been reported to induce angiogenesis and osteogenic differentiation simultaneously with NPs in MSCs.

Here, we have synthesized Zn-based iron oxide nanoparticles (ZIOs) that can release Zn and Fe ions in intracellular manner after endocytosis. We have synthesized bimetallic oxide NPs in distilled water, at relatively low temperature and pressure to deliver ions into stem cells for the first time. The ZIOs that can release two ions at the same time were expected to promote osteogenic differentiation of human MSCs (hMSCs) by Zn ions and angiogenic paracrine factor secretion from hMSCs by Fe ions. Through the cytotoxicity tests according to the concentration of ZIOs and gene expression tests for osteogenesis and angiogenesis the method for ZIO treatment to hMSCs was optimized. The results demonstrated in our study might support future tissue engineering for bone regeneration based on dual ion delivery system.

## 2. Materials and Methods

### 2.1. Materials

Zinc (II) chloride hexahydrate (98%, Zn(NO_3_)_2_·6H_2_O), iron (II) chloride (98%, FeCl_2_), polyethyleneglycol (Mn 20000, PEG), sodium hydroxide (97%, NaOH), and hydrochloric acid (37%, HCl) were purchased from Sigma Aldrich (St. Louis, MO, USA). There was no further purification process.

### 2.2. Synthetic Method of ZIOs

1 mmol of Zn(NO_3_)_2_·6H_2_O (0.2974 g), 1 mmol of FeCl_2_ (0.1267 g), 0.4 g of PEG, and 1.2 mmol of NaOH (0.048 g) were evenly dissolved in 2, 2, 5, and 1 mL of distilled water, respectively. Zn(NO_3_)_2_·6H_2_O, FeCl_2_, and PEG solutions were first mixed at room temperature. After 5 min stirring, the NaOH solution was injected into the reacting solution. The solution was then heated at 90 °C for 16 h. After 16 h heating, 0.2 mL of HCl was added. The product was centrifuged and washed twice with acetone (11,000 rpm, 10 min) and re-dispersed in distilled water.

### 2.3. Characterization

Transmission electron microscope (JEOL, JEM-2100F, Tokyo, Japan) equipped with an energy dispersive X-ray (EDS) were employed to analyze the nanostructure, morphology, and composition of the as-prepared nanoparticles. Powder X-ray diffraction (XRD) (Bruker, MA, USA) was conducted using a Rigaku D/MAX-2200PC X-ray diffractometer with Cu-Kα radiation (λ = 0.154 nm) at a scan rate of 6°/min. The chemical states of the as-prepared nanoparticles were investigated by X-ray photoelectron spectroscopy (XPS, ULVAC PHI, PHI 5000 VersaProbe, Osaka, Japan). The dissolution amount of nanoparticles was measured via inductively coupled plasma (ICP) (LEEMAN, Direct Reading Echelle ICP, OH, USA) spectrometer.

### 2.4. Cell Culture

hMSCs were purchased (Lonza, Walkersville, MD, USA) and cultured in 150-mm culture dishes using Dulbecco’s Modified Eagle Medium (DMEM; Gibco BRL, Gaithersburg, MD, USA) supplemented with 10% (*v*/*v*) FBS (Gibco BRL) and 1% (*v*/*v*) PS (Gibco BRL) in a 5% CO_2_ incubator at 37 °C. The culture medium was changed every other day. hMSCs with five to seven passages were used for the experiments. The NPs were mixed in a serum-free DMEM supplemented with 1% (*v*/*v*) PS (concentration from 0 to 40 μg/mL). The NPs were treated to hMSCs 24 h for a short period, and 72 h for a long period.

### 2.5. Neutral Red Assay and Live/Dead Assay

Cell cytotoxicity was evaluated using the neutral red (NR) assay and live/dead assay. hMSCs were seeded in 24-well plates (2 × 10^4^ cells/well) and incubated for 24 h. At 24 and 72 h after treated with ZIOs, the cells were washed with phosphate buffered saline (PBS; Gibco BRL), and were incubated with NR solution (0.005% (*w*/*v*), Sigma Aldrich) for an additional 2–3 h at 37 °C. The cells were washed with PBS and the addition of elution medium (50% EtOH and 1% acetic acid) was followed by gentle shaking for 10 min. The optical density (OD) of each well was recorded at 540 nm using a microplate reader (Infinite F50, Tecan, Mannedorf, Switzerland). Live/dead assay was performed using fluorescein diacetate (FDA; Sigma) and ethidium bromide (EB; Sigma). FDA (green) stains the cytoplasm of viable cells, whereas EB (red) stains the nuclei of nonviable cells. The staining solution was freshly prepared by mixing 10 mL of FDA stock solution (1.5 mg/mL of FDA in dimethyl sulfoxide), 5 mL of EB stock solution (1 mg/mL of EB in PBS), and 3 mL of PBS. Cells were then incubated with the staining solution for 3–5 min at 37 °C. After staining, the samples were washed twice or thrice with PBS and examined using a fluorescence microscope (DFC 3000 G, Leica, Wetzlar, Germany).

### 2.6. TUNEL Staining

Cellular viability was determined using the terminal deoxynucleotide transferase-mediated deoxyuridine triphosphate nick end labeling (TUNEL) staining. TUNEL staining was performed using an ApopTag^®^ Fluorescein In Situ Apoptosis Detection Kit (Millipore, Billerica, MA, USA) according to the manufacturer’s protocol. The cells were counter stained with 4,6-diamidino-2-phenylindole (DAPI; Vector Laboratories, Burlingame, CA, USA) and examined under a fluorescence microscope (DFC 3000 G, Leica, Wetzlar, Germany).

### 2.7. Quantitative Real Time Polymerase Chain Reaction (qRT-PCR)

Total RNA was extracted from the samples using 1 mL TRIzol (Ambion, Austin, TX, USA) and 200 μL of chloroform (Sigma). The samples were centrifuged at 12,000 rpm for 10 min at 4 °C. The RNA pellet was washed with 75% (*v*/*v*) ethanol (Sigma) in water and dried. After drying, the samples were dissolved in RNase-free water (iNtRON Biotechnology, Seoul, Korea). Reverse transcription was performed using 1.5 μg of pure total RNA and Primescript RT master Mix (TaKaRa, Kusatsu, Japan), followed by PCR amplification of the synthesized cDNA. For qRT-PCR, the SsoAdvanced Universal SYBR Green Supermix (Bio-Rad, Hercules, CA, USA) and the CFX Connect™ real-time PCR detection system (Bio-Rad) was used. For the in vitro assay, qRT-PCR was used to quantify the relative expression of *BAX*, *BCL-2*, *RUNX2*, *BMP-2*, *COL 1*, *OSTERIX*, *ALP*, and *VEGF*. *GAPDH* served as the internal control. The sequences of primers used for qRT-PCR are listed in Table 1.

### 2.8. F-Actin Staining

The hMSCs were seeded in 6 well plates (0.7 × 10^5^ cells/well) and incubated for 24 h. At 72 h after the ZIOs had been treated, the cells were washed with PBS (Gibco BRL) and then the cells were fixed with 4% paraformaldehyde (Biosesang, Sungnam, Korea) in PBS for 10 min at room temperature. For the phalloidin staining, the fixed hMSCs were stained with TRITC-phalloidin containing a mounting medium (VECTASHIELD H-1600, Vector, Burlingame, CA, USA), counter-stained with 4′,6-diamidino-2-phenylindole (DAPI, Vector), and then examined using a fluorescence microscope (DFC 3000 G, Leica, Wetzlar, Germany).

### 2.9. Alkaline Phosphatase (ALP) Staining

ALP activity in the cells was identified using the ALP stain kit (MK300, Takara, Kusatsu, Japan). Following 72 h of treatment, the cells were fixed with fixation solution, and stained with substrate solution for ALP, according to the manufacturer’s protocol. After staining, the samples were examined using a microscope (CKX53, Olympus, Tokyo, Japan).

### 2.10. Enzyme-Linked Immunosorbent Assay (ELISA)

Cell supernatants were collected at 24 h or 72 h after treatment with the NPs. The concentration of cytokines was measured using ELISA kits for human VEGF (R&D system, Minneapolis, MN, USA) according to the manufacturer’s protocol.

### 2.11. Reactive Oxygen Species (ROS) Staining

ROS levels were measured using 2′,7′-dichlorodihydrofluorescein diacetate (DCF; D339 Invitrogen, Carlsbad, CA, USA), a fluorescent indicator of ROS. hMSCs treated with or without NPs, were incubated with 10 μM DCF prepared in PBS for 20 min at 37 °C. After staining, the samples were washed twice with PBS and examined under a fluorescence microscope (DFC 3000 G, Leica, Wetzlar, Germany). The intracellular ROS concentration was also evaluated by fluorescence intensity (Ex/Em of 494 nm/524 nm) using a microplate reader (Varioskan LUX multimode microplate reader, Thermo Fisher Scientific, Waltham, MA, USA).

### 2.12. Statistical Analysis

All quantitative data are expressed as mean ± standard deviation (SD). Statistical analysis was performed using the Student’s *t*-test or the one-way ANOVA using a Bonferroni test; *p* < 0.05 was considered significant.

## 3. Results and Discussion

### 3.1. Characterization of ZIOs

**Figure 1:**
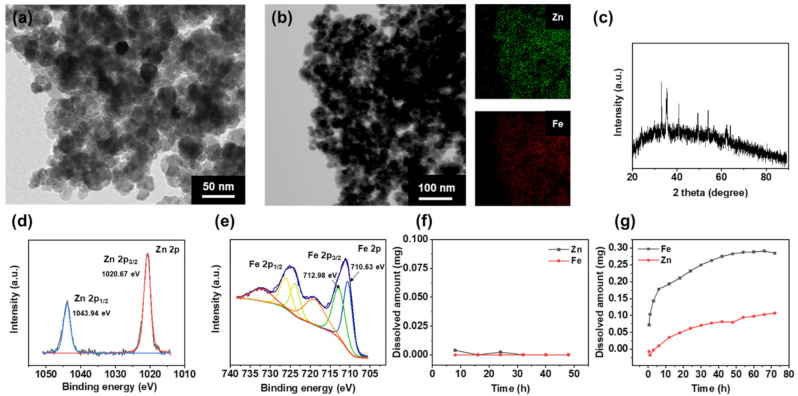
Characterization of the Zn-based iron oxide nanoparticles (ZIOs). (**a**) The representative transmission electron microscopy (TEM) image and (**b**) energy dispersive spectroscopy (EDS) elemental mapping image with (**c**) the X-ray diffraction (XRD) pattern of Zn-based IONs. High resolution XPS spectra of (**d**) Zn 2p and (**e**) Fe 2p. (**f**) Zn, Fe ion dissolution graph of the ZIOs in pH 7 controlled solution. (**g**) Cumulative release profiles of Zn, Fe ions from ZIOs under a pH 4.5 condition.

ZIOs were synthesized by reacting Zn, Fe precursors with NaOH in an aqueous-phase in the presence of polyethylene glycol (PEG) as a stabilizer. Transmission electron microscopy (TEM) and energy dispersive spectroscopy (EDS) analyses showed that the synthesized ZIOs had spherical morphology with 20~40 nm size and a uniform dispersion of Zn and Fe in the NPs (Figure 1a,b). X-ray diffraction (XRD) patterns of the NPs shown in Figure 1c indicate the presence of ZnFe_2_O_4_ (29.94°, 35.22°, 42.9°, 56.53°, and 62.34°, JCPDS file No. 89-1010) and α-Fe_2_O_3_ (24.24°, 33.11°, 35.61°, 40.81°, 49.37°, 53.96°, 63.91°, and 71.66° JCPDS file No. 33-0664).

Since ZnFe_2_O_4_ and Fe_3_O_4_ show similar XRD patterns, the detailed crystal structure of the ZIOs, and the oxidation state of Zn and Fe in the NPs were analyzed using X-ray photoelectron spectroscopy (XPS). The Zn 2p XPS spectrum includes Zn 2p_3/2_ and Zn 2p_1/2_ peaks at 1020.67 and 1043.94 eV, respectively, which corresponds to Zn^2+^ (Figure 1d) [20]. In the Fe 2p XPS spectrum shown in Figure 1e, the Fe^2+^ (710.63 eV) peak and the Fe^3+^ (712.98 eV) peak were confirmed. These results revealed that ZIOs are composed of ZnFe_2_O_4_, Fe_3_O_4_, and α-Fe_2_O_3_.

Dissolution of ZIOs was treated in a weak acidic condition (pH 4.5) and neutral condition (pH 7.0), respectively. The dissolution amount of Zn and Fe was characterized by inductively coupled plasma (ICP). In the neutral condition, the ZIOs were hardly dissolved (Figure 1f). When we decreased pH to 4.5, which is a similar condition in endosome, ZIOs were dissolved and started to release Zn and Fe ions (Figure 1g).

### 3.2. Cytotoxicity According to ZIOs Concentration on hMSCs

**Figure 2:**
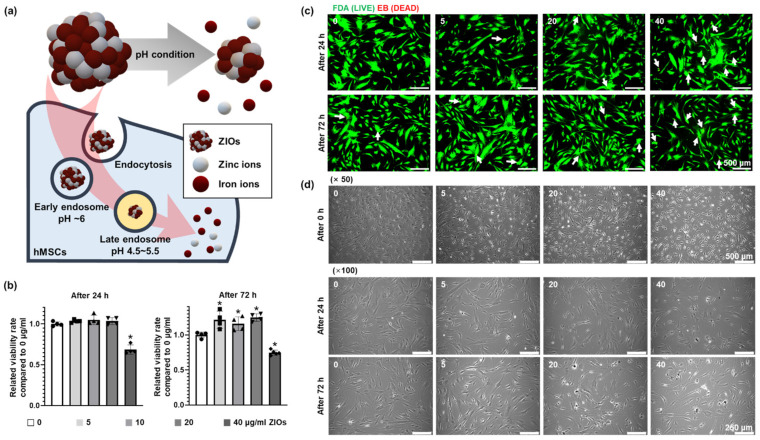
Cytotoxicity of ZIOs on human mesenchymal stem cells (hMSCs) according to ZIOs concentration and treating time. (**a**) Schematic diagram depicting the endocytosis of the ZIOs to hMSCs. To determine the cytotoxicity of the ZIOs on hMSCs, (**b**) relative cell viability ratio was evaluated by neutral red assay using the 0 μg/mL group (normal cells) as a control (* *p* < 0.05, compared to the control group). (**c**) Apoptotic activity of hMSCs after ZIOs treatment as evaluated by the fluorescein diacetate ethidium bromide (FDA-EB) assay (live cells; green, dead cells; red). Dead cells were indicated with white arrows. (Scale bars: 500 μm). (**d**) Representative cell morphology of hMSCs after ZIOs treatment (Scale bar: 500 μm (×50) and 250 μm (×100)).

Figure 2a shows a schematic for the method of applying ZIOs to hMSCs. When the ZIOs were delivered into cells through endocytosis, the ZIOs were exposed to the low pH environment of the endosome [13]. Thereafter, the ZIOs underwent degradation into Zn and Fe ions. The ZIOs were mixed in a serum-free medium to prevent aggregation with the serum before treating to hMSCs [21,22]. To determine the cytotoxicity of the ZIOs on hMSCs, the ZIOs were treated with different concentrations (0 to 40 μg/mL) for 24 h or 72 h. Results from neutral red (NR) assay indicated that the 40 μg/mL of NPs for both 24 h and 72 h treatment showed significant cytotoxicity in hMSCs compared to other groups (Figure 2b). Fluorescent staining images of live/dead cells showed that dead cells (red) were also increased significantly in 40 μg/mL group compared to the other groups (Figure 2c). Morphological difference in hMSCs after the ZIOs treatments was not observed in 0, 5, 10, and 20 μg/mL groups in contrast to 40 μg/mL group (Figure 2d). We have confirmed that treating ZIOs up to 20 μg/mL concentration to hMSCs did not induce cytotoxicity.

### 3.3. Effects of ZIOs on hMSCs Viability

**Figure 3:**
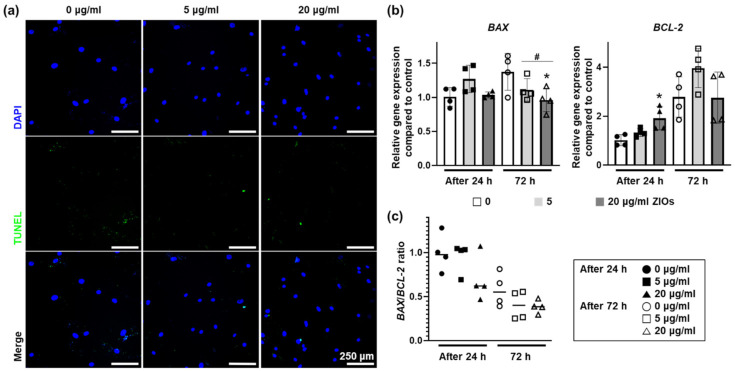
Effects of ZIOs on hMSCs viability. The cellular viability of the hMSCs treated with the ZIOs as evaluated by (**a**) TUNEL staining (72 h, green indicates dead cells, blue indicates nuclear, scale bars indicate 250 μm) and (**b**) pro-apoptotic (*BAX*) and anti-apoptotic (*BCL-2*) gene expression at 24 and 72 h (* *p* < 0.05, compared to the 0 μg/mL group, # *p* < 0.05, compared to each group). (**c**) *BAX/BCL-2* ratio in hMSCs at 24 and 72 h after the ZIOs treatments.

We examined the apoptotic effects of ZIOs on hMSCs after treating 5 or 20 μg/mL of ZIOs to hMSCs for 24 or 72 h. After treatments, there were no significant differences in terminal deoxynucleotidyl transferase dUTP nick end labeling positive (TUNEL^+^) cells in 5 and 20 μg/mL groups as compared to the control group (0 μg/mL, Figure 3a). To investigate whether the ions released from the ZIOs could trigger cell death, the expression of apoptosis related genes was evaluated via quantitative real time polymerase chain reaction (qRT-PCR). The expression of *BAX*, pro-apoptotic gene, decreased significantly in both 5 and 20 μg/mL groups on 72 h compared to the control group (Figure 3b). The expression of *BCL-2*, an anti-apoptotic gene, increased significantly in the 20 μg/mL groups on 24 h (Figure 3b). However, the *BAX/BCL-2* ratio in hMSCs after the ZIOs treatments showed that there were no statistical differences between 5 and 20 μg/mL groups (Figure 3c). That is, the ZIOs did not affect DNA damage or gene expression that related with cell death on hMSCs up to 20 μg/mL treatment.

### 3.4. Effect of Zn Ions Released from ZIOs on Osteogenic Differentiation of hMSCs

**Figure 4:**
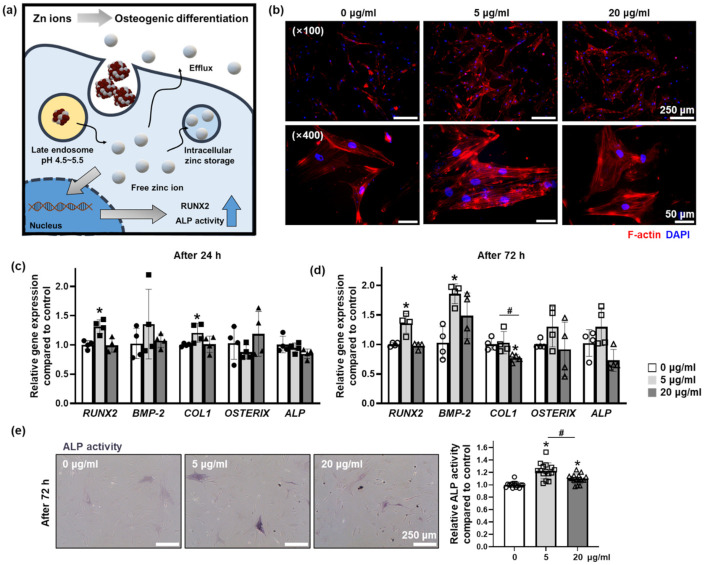
Effect of Zn ions released from ZIOs on osteogenic differentiation of hMSCs. (**a**) Schematic illustration describing the effect of Zn ions on hMSCs to osteogenic differentiation. (**b**) Fluorescence images of F-actin (red) in the hMSCs after the ZIOs treatment (72 h). The nuclei were stained with DAPI (blue). Scale bars indicate 250 μm (×100) and 50 μm (×400). Expression of osteogenic differentiation related genes, *Runx2*, bone morphogenetic protein 2 *(BMP-2)*, collagen 1 *(COL1)*, *OSTERIX*, and alkaline phosphatase *(ALP)* after treating the ZIOs for (**c**) 24 h or (**d**) 72 h (* *p* < 0.05, compared to the control group (0 μg/mL group)). (**e**) Representative images of ALP staining (left, purple) in the hMSCs at 72 h. Scale bars indicate 250 μm. The ratio of the relative ALP activity in hMSCs after the ZIOs treatment (right, 72 h) (* *p* < 0.05, compared to the control group, # *p* < 0.05, compared to each group).

The Zn and Fe ions safely delivered to hMSCs in intracellular manner through the endocytosis of the ZIOs played a critical role to modulate cellular behaviors. The Zn ions delivered into hMSCs with our ZIOs promoted early osteogenic differentiation of hMSCs (Figure 4a). The F-actin staining results showed that the highest F-actin expression was found in the 5 μg/mL group compared to other groups (Figure 4b). It has been reported that Zn ions could increase the expression of F-actin in the cells [23]. Additionally, upregulated F-actin expression has been reported to induce osteogenic differentiation of stem cells [24,25]. We found that the expression of *RUNX2* in hMSCs was significantly increased in 5 μg/mL groups compared to other groups both after 24 h (Figure 4c) and 72 h (Figure 4d). In addition to *RUNX2* expression, we observed that expressions of bone morphogenetic protein 2 (*BMP-2*, Figure 4d) and collagen 1 (*COL1*, Figure 4c), which are representative early osteogenic differentiation markers, were significantly increased in 5 μg/mL group compared to the control group (0 μg/mL). On the other hand, the expression of *COL1* in 20 μg/mL group at 72 h was decreased compared to other groups (Figure 4d). There was no statistical difference in the late osteogenic marker expression, *OSTERIX* and alkaline phosphatase (*ALP*) in hMSCs after ZIOs treatment within 72 h (Figure 4c,d). However, ALP staining results showed that the highest ALP activity was detected in the 5 μg/mL group compared to other groups (Figure 4e). It has been reported that Zn ions are essential to bone differentiation and bone tissue development [26]. Zinc ions also act as signaling molecules, affecting intracellular signaling pathways [27]. High concentrations of Zn ions not only decrease bone differentiation capacity, but also induce apoptosis [28,29]. However, moderate amounts of Zn ions can promote osteogenic differentiation of stem cells through upregulation of the ERK pathway, especially increasing the expression of RUNX2 [16,17]. In addition, similar to previous studies, Zn ions promoted osteogenic differentiation of stem cells when the concentration of Zn ions was less than 20 μg/mL [28,30]. Therefore, we have concluded that Zn ions delivered through the ZIOs promoted the early osteogenic gene expression and osteogenic differentiation capacity of hMSCs when treated with 5 μg/mL of ZIOs within 3 days. The osteogenic differentiation of hMSCs has been induced through an osteogenic differentiation medium or growth factor such as BMP-2 [31,32,33]. Compared to conventional materials, the ZIOs used in this study have advantages such as temperature independent bioactivity and low cost. Additionally, ZIOs can be applied to future in vivo tissue regeneration since the ZIOs have high response to external magnetic force (Figure S1, Supplementary Materials), which has been used to enhance the therapeutic effect of stem cells [34].

### 3.5. Effect of Fe Ions Released from ZIOs on Angiogenic Paracrine Factors Secretion from hMSCs

**Figure 5:**
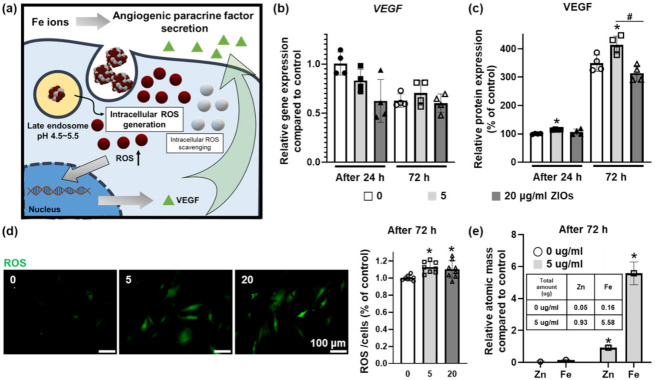
Effect of Fe ions released from ZIOs on angiogenic paracrine factors secretion in hMSCs. (**a**) Schematic illustration describing the effect of Fe ions to hMSCs in angiogenic paracrine factors secretion. (**b**) Relative expression of vascular endothelial growth factor (*VEGF*) evaluated by quantitative real time polymerase chain reaction (qRT-PCR). (**c**) Secretion of VEGF from hMSCs was evaluated by in enzyme-linked immunosorbent assay (ELISA) (* *p* < 0.05, compared to the control group (0 μg/mL), # *p* < 0.05, compared with each group). (**d**) Staining of intracellular ROS using DCF-DA (green), and its quantification (* *p* < 0.05, compared to the control group (0 μg/mL)). (**e**) Relative atomic mass in the hMSCs compared to control group evaluated with ICP-MS (* *p* < 0.05, compared to the control group (0 μg/mL)).

The Fe ions are known to increase the secretion of angiogenic paracrine factors in stem cells by raising the ROS level in the cells [35]. On the contrary, the Zn ions are known to exhibit antioxidant effect in the cells [36,37,38] (Figure 5a). There was no significant difference in the gene expression of *VEGF* from hMSCs treated with ZIOs compared to control group (0 μg/mL group, Figure 5b). However, as shown in enzyme-linked immunosorbent assay (ELISA) results, the concentration of VEGF secreted from hMSCs was highest at the 5 μg/mL groups (Figure 5c). When checking the intracellular ROS level, higher ROS values were detected in both 5 and 20 μg/mL groups compared to the control group. However, there was no statistical difference in ROS expression by concentration difference of the ZIOs (Figure 5d). This might be caused by the Zn ions that play the role of ROS scavenging. Through ICP analysis, the amount of the ZIOs remaining in the cells was confirmed at 5 μg/mL in groups based on the amount of Zn and Fe as shown in the Figure 5e. Although the high concentration of metal ions can induce cell death, an appropriate concentration of ions can stimulate specific cellular behaviors. Treating appropriate concentration of Fe ions to stem cells has been reported to promote the secretion of angiogenic paracrine factors by increasing the expression of VEGF through ROS modulation; Zn ions usually do not undergo redox reaction directly due to their stability. However, Zn ions can induce antioxidant reactions in the cells indirectly [36,37,38,39]. Zn ions are also known to affect transcriptional factors such as nuclear factor erythroid 2-related factor 2 or to increase the synthesis of metallothionein, which acts as an oxidant scavenger resulting in the ROS scavenging effect [36,38]. As reported previously, intracellular ion homeostasis can affect the cell viability [40] and the balance among intracellular ions [41]. For example, high intracellular concentration of Zn ions has been reported to induce ferroptosis, Fe ion-mediated cell death, through disrupting intracellular Fe ions concentration. If the Zn ion concentration increases rapidly in the cells, Fe mediated metabolisms undergo disruption and thus, cause Fe overload in the cells that can induce ferroptosis [41]. In order to enhance the therapeutic efficacy of stem cells through intracellular ions delivery, the concentration of ions must be elaborately adjusted to avoid any disruption in cellular ion homeostasis.

## 4. Conclusions

In this study, we have synthesized bimetallic ZIOs under relatively mild experimental conditions and used them for biomedical applications. ZIOs were introduced to hMSCs to modulate the osteogenic differentiation. The synthesized ZIOs dissolved under weak acid conditions which mimics the pH of the endosome condition. Through our dual ion releasing ZIOs, the Zn and Fe ions were successfully delivered to hMSCs without cytotoxicity and enhanced the osteogenic differentiation and angiogenic paracrine factor secretion. The results demonstrated in our study might support the future tissue engineering for bone regeneration based on dual ion delivery system.

## Figures and Tables

**Table 1:** Quantitative real time polymerase chain reaction (qRT-PCR) primer sequences.

Gene	Primer	Sequence (5′–3′)
*Human GAPDH*	Forward	GTC GGA GTC AAC GGA TTT GG
	Reverse	GGG TGG AAT CAA TTG GAA CAT
*Human BAX*	Forward	GCA ACT TCA ACT GGG GCC GGG
	Reverse	GAT CCA GCC CAA CAG CCG CTC
*Human BCL-2*	Forward	CAA CAT CGC CCT GTG GAT GA
	Reverse	GGG CCA AAC TGA GCA GAG TC
*Human RUNX2*	Forward	TCA CTA CCA GCC ACC GAG AC
	Reverse	ACG CCA TAG TCC CTC CTT TT
*Human BMP-2*	Forward	TGT ATC GCA GGC ACT CAG GTC A
	Reverse	CCA CTC GTT TCT GGT AGT TCT TC
*Human COL 1*	Forward	TGC GAT GAC GTG ATC TGT GA
	Reverse	TTG GTC GGT GGG TGA CTC TG
*Human OSTERIX*	Forward	TAA TGG GCT CCT TTC ACC TG
	Reverse	CAC TGG GCA GAC AGT CAG AA
*Human ALP*	Forward	CCT CCT CGG AAG ACA CTC TG
	Reverse	GCA GTG AAG GGC TTC TTG TC
*Human VEGF*	Forward	GAG GGC AGA ATC ATC ACG AAG T
	Reverse	CAC CAG GGT CTC GAT TGG AT

## Data Availability

The data presented in this study are available on request from the corresponding author.

## Supplementary Materials

The following are available online at https://www.mdpi.com/1996-1944/14/2/412/s1, Figure S1: Magnetic property of the Zn-based iron oxide nanoparticles (ZIOs).
