# Supplementary material for: Dual Ion Releasing Nanoparticles for Modulating Osteogenic Cellular Microenvironment of Human Mesenchymal Stem Cells
Source: Materials (Basel). 2021 Jan 15;14(2):412. doi: 10.3390/ma14020412 (PMC7830414; doi:10.3390/ma14020412)
Supplement: Supplementary file 1 [file materials-14-00412-s001.pdf]

materials

Supplementary Materials

Dual Ion Releasing Nanoparticles for Modulating Osteogenic Cellular Microenvironment of Human Mesenchymal Stem Cells

Yu-Jin Kim [1,†], Jaeyoung Lee [2,†], Gwang-Bum Im [1], Jihun Song [1], Jiwoo Song [2,3], Jiyong Chung [2,3,*], Taekyung Yu [2,3,*] and Suk Ho Bhang [1,*]

[1] School of Chemical Engineering, Sungkyunkwan University, Suwon 16419, Korea; yujinkim1003@gmail.com (Y.-J.K.); lki1005@skku.edu (G.-B.I.); jih2616@naver.com (J.S.)
[2] Department of Chemical Engineering, Kyung Hee University, Youngin 17104, Korea; skvneu30@gmail.com (J.L.); jiwoo715@naver.com (J.S.)
[3] BK21 FOUR Integrated Engineering Program, Department of Chemical Engineering, Kyung Hee University, Youngin 17104, Korea
* Correspondence: jychung12@khu.ac.kr (J.C.); tkyu@khu.ac.kr (T.Y.); sukhobhang@skku.edu (S.H.B.); Tel.: +82-31-201-2862 (J.C.); +82-31-204-8114 (T.Y.); +82-31-290-7242 (S.H.B.)
† These authors contributed equally to this work.

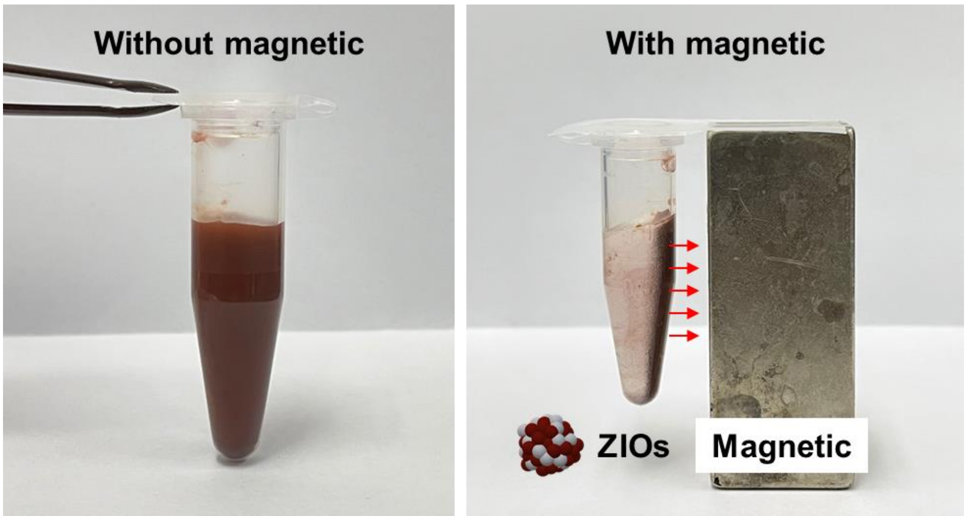

Figure S1. Magnetic property of the Zn-based iron oxide nanoparticles (ZIOs).

Publisher's Note: MDPI stays neutral with regard to jurisdictional claims in published maps and institutional affiliations.

Copyright: © 2021 by the authors. Submitted for possible open access publication under the terms and conditions of the Creative Commons Attribution (CC BY) license (http://creativecommons.org/licenses/by/4.0/).
